# Factors Associated with Disparities in Appropriate Statin Therapy in an Outpatient Inner City Population

**DOI:** 10.3390/healthcare8040361

**Published:** 2020-09-24

**Authors:** Giselle Alexandra Suero-Abreu, Aris Karatasakis, Sana Rashid, Maciej Tysarowski, Analise Douglas, Richa Patel, Emaad Siddiqui, Aishwarya Bhardwaj, Christine M. Gerula, Daniel Matassa

**Affiliations:** 1 Department of Medicine, Rutgers New Jersey Medical School, Newark, NJ 07103, USA; aris.karatasakis@gmail.com (A.K.); rashidsana50@gmail.com (S.R.); m.tysarowski@rutgers.edu (M.T.); analiseydouglas@gmail.com (A.D.); dan.matassa@rutgers.edu (D.M.); 2 Department of Medicine, Division of Cardiology, University of Washington, Seattle, WA 98195, USA; 3 Division of Cardiology, Department of Medicine, University of Connecticut, Hartford Hospital, Hartford, CT 06102, USA; 4 Department of Medicine, Icahn School of Medicine at Mount Sinai, New York, NY 10029, USA; richa.patel@mountsinai.org; 5 Department of Medicine, NYU Langone Medical Center, New York, NY 10016, USA; emaad.siddiqui@nyulangone.org; 6 Division of Cardiology, Department of Medicine, Rutgers New Jersey Medical School, Newark, NJ 07103, USA; ab1898@njms.rutgers.edu (A.B.); gerula@njms.rutgers.edu (C.M.G.)

**Keywords:** hyperlipidemia, statin, lipid-lowering agents, adherence, healthcare disparities

## Abstract

Lipid-lowering therapies are essential for the primary and secondary prevention of atherosclerotic cardiovascular disease (ASCVD). The aim of this study is to identify discrepancies between cholesterol management guidelines and current practice with a focus on statin treatment in an underserved population based in a large single urban medical center. Among 1042 reviewed records, we identified 464 statin-eligible patients. Age was 61.0 ± 10.4 years and 53.9% were female. Most patients were black (47.2%), followed by Hispanic (45.7%) and white (5.0%). In total, 82.1% of patients were prescribed a statin. An appropriate statin was not prescribed in 32.4% of statin-eligible patients who qualified based only on a 10-year ASCVD risk of ≥7.5%. After adjustment for gender and health insurance status, appropriate statin treatment was independently associated with age >55 years (OR = 4.59 (95% CI 1.09–16.66), *p* = 0.026), hypertension (OR = 2.38 (95% CI 1.29–4.38), *p* = 0.005) and chronic kidney disease (OR = 3.95 (95% CI 1.42–14.30), *p* = 0.017). Factors independently associated with statin undertreatment were black race (OR = 0.42 (95% CI 0.23–0.77), *p* = 0.005) and statin-eligibility based solely on an elevated 10-year ASCVD risk (OR = 0.14 (95% CI 0.07–0.25), *p* < 0.001). Hispanic patients were more likely to be on appropriate statin therapy when compared to black patients (86.8% vs. 77.2%). Statin underprescription is seen in approximately one out of five eligible patients and is independently associated with black race, younger age, fewer comorbidities and eligibility via 10-year ASCVD risk only. Hispanic patients are more likely to be on appropriate statin therapy compared to black patients.

## 1. Introduction

Atherosclerotic cardiovascular disease (ASCVD) is a leading cause of morbidity and mortality globally, with over 600,000 deaths in the United States annually [1,2]. Statins are an effective therapy for the primary and secondary prevention of ASCVD, with proven mortality benefits and robust long-term safety data [3]. Reflecting this, current ASCVD prevention guidelines, including the American Heart Association and American College of Cardiology (AHA/ACC) multisociety guidelines on the management of blood cholesterol, have recommended statins as the cornerstone of lipid-lowering therapy (LLT) for primary and secondary ASCVD prevention [4]. Nonetheless, gaps exist in guideline-directed statin prescription patterns in clinical practice. For example, racial and ethnic minorities and patients with socioeconomic barriers to healthcare face disparities that result in worse outcomes and higher mortality rates secondary to ASCVD [5]. Younger black patients are particularly vulnerable to these disparities in statin prescription patterns [6]. This effect of age and race on statin prescription, compounded by a general delay in the adoption of new guidelines by healthcare providers, leads to suboptimal patient care in these populations [7]. The objectives of this study were (1) to identify discrepancies between cholesterol management guidelines and current practice in an inner city academic center primary care population; and (2) to provide insights into possible interventions aimed at improving statin prescribing rates and thus reducing the incidence of ASCVD in this vulnerable cohort.

## 2. Materials and Methods

**Figure 1:**
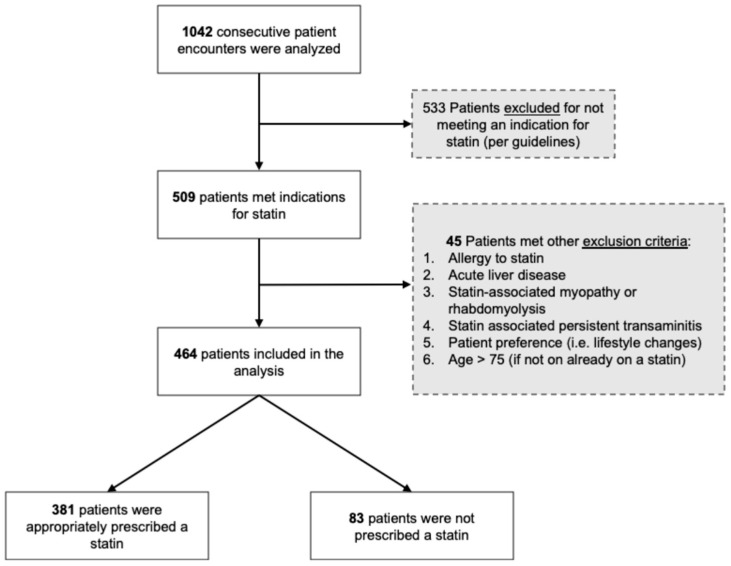
Flow diagram with patient selection and study criteria.

We retrospectively analyzed 1042 consecutive patient encounters taking place between August 2018 and August 2019 at the University Hospital Ambulatory Care Center, Newark, NJ, USA, which is a large single center located in an urban area with a predominant underserved minority population where 54.7% of the patients are without health insurance. Compared to the rest of the state of New Jersey and the country, the city of Newark has a higher rate of diabetes, coronary artery disease, stroke and chronic kidney disease. We determined eligibility for statins and other LLT in adults aged 20–75 years based on the 2018 AHA/ACC multisociety guidelines on the management of blood cholesterol. We identified 509 statin-eligible patients, out of which 464 patients were included in the final analysis; 45 patients met the exclusion criterion of well-documented contraindications to statins (e.g., allergy, patient preference/hesitancy or side effects) and were removed from the analysis (Figure 1). High-intensity statins were defined as Atorvastatin 40–80 mg and Rosuvastatin 20–40 mg. All data were obtained from the electronic health record and this study was approved by the Institutional Review Board (Pro2018002828). The study met the requirements of the Declaration of Helsinki and was performed in compliance with human studies guidelines. Individual consent for participation in anonymous data analysis was waived. Data were collected and managed via Research Electronic Data Capture (REDCap), a secure web-based software platform designed to support data capture, hosted at Rutgers New Jersey Medical School [8]. The pooled cohort equation was used to estimate ASCVD risk and criteria for statin eligibility were determined based on guidelines current at the time of the patient encounter. Data are presented as mean ± standard deviation for normally distributed continuous variables or median (IQR=interquartile range) for non-normally distributed continuous variables and compared using the Wilcoxon rank-sum test. Categorical variables are presented as a number (percentage) and compared using the chi-square. Multivariate logistic regression analysis was performed to identify independent predictors of statin prescription in the cohort. Odds ratios (OR) were calculated to determine the association between patient characteristics and statin treatment status. All statistical analyses were two-sided and significance was established at α = 0.05. Analyses were performed using R statistical software version 3.6.1, R Foundation for Statistical Computing, Vienna, Austria.

## 3. Results

Among 464 statin-eligible patients, the average age was 61.0 ± 10.4 and the majority were female (53.9%, Table 1). The majority of patients identified as black (47.2%), followed by Hispanic or Latino (45.7%), white (5.0%), Asian (1.9%) and Pacific Islander (0.2%). Furthermore, 38.8% did not report English as their primary language, out of which 70.6% preferred translation services during the encounter. Most (54.7%) patients lacked health insurance. The most common comorbidities were hypertension (81.0%), diabetes mellitus (45.0%), chronic kidney disease (15.3%), cerebrovascular disease (14.7%), coronary artery disease (14.6%), heart failure (12.9%) and peripheral artery disease (2.8%); 17.9% of patients were current smokers. A lipid profile was available for 97.4% of patients and the median LDL-C was 87 mg/dl [IQR 64.0–120.0]. Among diabetic patients, the median hemoglobin A1c was 7.9% [IQR 6.5–8.9].

**Figure 2:**
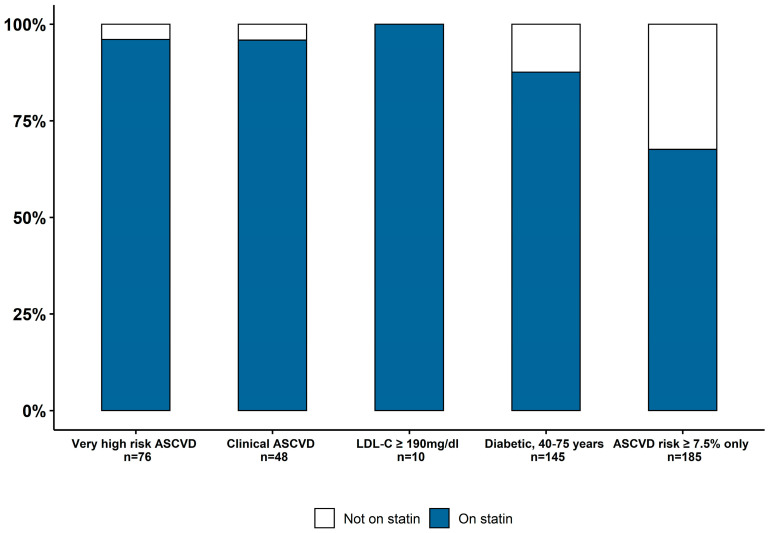
Rate of statin prescription and underprescription based on statin eligibility group per 2018 AHA/ACC guidelines.

Patients’ indication for statin was as follows (in hierarchical order): very high-risk ASCVD (16.4%, *n* = 76), clinical ASCVD (10.3% *n* = 48), LDL-C ≥190 mg/dl (2.2% *n* = 10), diabetes in 40–75 years old (31.3% *n* = 145), and 10-year ASCVD risk ≥7.5% (39.9% *n* = 185), and the majority (82.1%) of statin-eligible patients were prescribed a statin (Figure 2). A high-intensity statin was indicated in 69.6% of patients and was appropriately prescribed in 88.5% of these patients. Appropriate statin prescription was associated with the presence of common comorbidities such as diabetes, chronic kidney disease, heart failure, chronic kidney disease, heart failure, coronary artery disease, cerebrovascular diseases and lower diastolic blood pressure (Table 1, *p* < 0.05). The most commonly prescribed statins and doses were Atorvastatin 40–80 mg (61.2%), Atorvastatin 10–20 mg (9.7%), Pravastatin 40–80 mg (4.7%), Simvastatin 20–40 mg (4.5%) and Rosuvastatin 20–40 mg (0.5%). Of those already prescribed a statin, only 27.2% of patients were monitored with subsequent LDL-C ordered for treatment efficacy. Out of the 126 patients on statins who had inadequate LDL-C reduction at follow-up while on the maximally tolerated statin, 40.4% met criteria for Ezetimibe and 8.7% met criteria for PCSK9 inhibitors. Incidence of Ezetimibe prescription was only 22% and none of the patients that met criteria for PCSK9 inhibitor were prescribed this treatment. Persistent hypertriglyceridemia (defined as ≥175 mg/dl for three or more measurements) was identified in 3.2% of patients on high-intensity statins, while only 0.21% were prescribed a triglyceride-lowering therapy. The physicians who treated the patients were internal medicine residents at postgraduate level one to three that were supervised by general medicine attending physicians. In a univariate analysis, there was no difference in LLT prescribing patterns when looking at prescribers at different levels of training experience between one and three years. Approximately one third (30.4%) of patients had an outpatient cardiology visit within 12 months of the index encounter, out of which 92.2% (130 patients *p* < 0.0001) were placed on an appropriate statin. This could suggest that general medicine practitioners were possibly underprescribing statins to certain groups, but a multivariate analysis did not confirm this finding and further study is needed to explore this finding.

**Figure 3:**
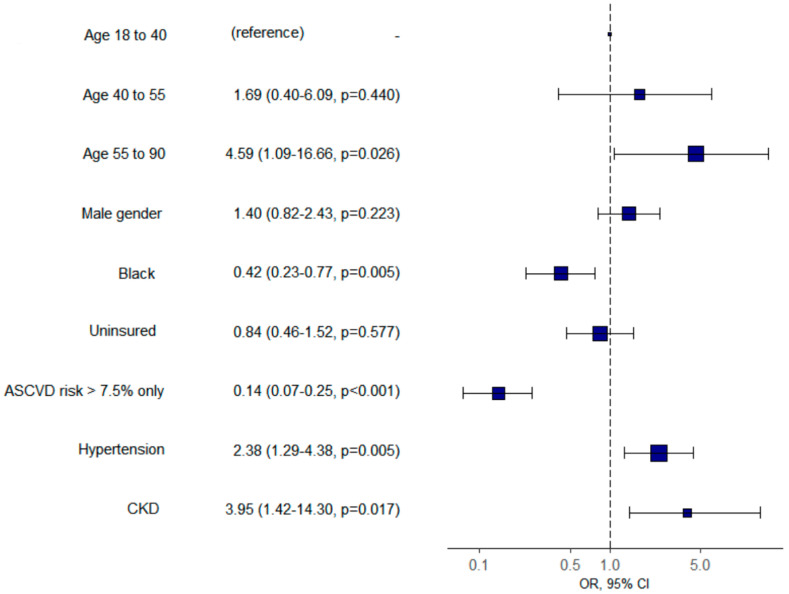
Adjusted odds of statin prescribing by age (reference age 18–40 years old) for various comorbidities (95% CI, *p*-value).

As compared with statin-eligible patients appropriately treated with statins, eligible patients who were not prescribed a statin were more likely to be black (60.2% vs. 44.9%, *p* < 0.0001), younger (57.2 years old ± 9.1 vs. 61.8 ± 10.5, *p* = 0.0001) and female (55.4%). The majority (72.3%) of statin-eligible patients not placed on statins were indicated solely based on a calculated 10-year ASCVD of ≥7.5%, as compared with 32.8% in the appropriately treated group (*p* < 0.0001). Among this group, 32.4% did not have a statin prescribed; this was therefore the set of patients with the lowest prescribing rate among all statin benefit groups In a multivariate analysis, after adjustment for gender and insurance status, appropriate statin treatment correlated positively with older age (OR = 4.59 (95% CI 1.09–16.66), *p* = 0.026), hypertension (OR = 2.38 (95% CI 1.29–4.38), *p* = 0.005) and chronic kidney disease (OR = 3.95 (95% CI 1.42–14.30), *p* = 0.017) (Figure 3). Race was a significant predictor of statin prescribing and black patients were less likely to receive a statin (OR = 0.42 (95% CI 0.23–0.77), *p* = 0.005). The negative correlation was similar for the 10-year ASCVD risk ≥7.5% only benefit group (OR = 0.14 (95% CI 0.07–0.25), *p* < 0.001) (Figure 3). Conversely, Hispanic patients were more likely to be on appropriate statin therapy when compared to black patients (86.8% vs. 77.2%). There was no association between appropriate statin therapy and health insurance status or the gender of the patient in our stepwise logistic regression model.

## 4. Discussion

The AHA/ACC cholesterol guidelines emphasize appropriate statin therapy and the monitoring of its efficacy in statin-eligible groups to reduce the risk of micro- and macrovascular complications related to dyslipidemia. Our study demonstrated that the majority of statin-eligible patients were prescribed a statin following best prescribing patterns for secondary prevention groups (e.g., very high-risk ASCVD, clinical ASCVD). However, most patients did not have subsequent LDL-C measurements to assess the effectiveness of statin therapy. This may be associated with some patients not being maximized on statin therapy and could have thus contributed to lower prescription of adjunct LLTs, such as Ezetimibe and PCSK-9 inhibitors.

In addition, younger patients were more likely to meet statin indication solely with 10-year ASCVD risk assessment of ≥7.5% and were also less likely to be prescribed a statin. In this context, underutilization of the 10-year ASCVD risk calculator could have led to the underpresciption of statins in younger patients with fewer comorbidities who met statin eligibility solely with an ASCVD risk of ≥7.5%. This shows that assessment of the 10-year ASCVD risk is paramount to early primary prevention in younger patients and that there is a need to re-stratify these patients based on risk enhancers according to the 2018 ACC/AHA guidelines. It is also noteworthy that since ASCVD manifests as early as the fifth decade of life, and given the proven mortality benefit of statins, early primary prevention is crucial to prevent CVD events and death, with each 1 mg/dL LDL-C reduction correlating to a 1% decrease in the risk of CVD [3,4,9].

In our study, the vast majority of the patient population was either black or Hispanic, with lower statin prescription rates noted in black patients. In particular, younger black patients were less likely to be on an appropriate statin, especially when the sole indication was a 10-year ASCVD risk score of ≥7.5%. Health disparities in black patients with CVD have previously been demonstrated in several studies [6,10,11]. Dorsch et al. conducted a retrospective study in 2019 with over 9000 participants which showed statin underprescription in younger black patients when compared with majority white patients. Similarly, the PALM (Patient and Provider Assessment of Lipid Management) registry found that black patients were underprescribed statins across multiple specialties when compared to white patients and the REGARDS (Reasons for Geographic and Racial Differences in Stroke) study cited lower rates of statin use by black patients living in poverty who lacked health insurance [11,12]. Our study expands on these data by comparing black patients to a largely Hispanic population. Although CVD is the leading cause of death among Hispanic patients, they remain underrepresented in studies [13]. When compared with black patients in our study, Hispanic patients had a higher statin prescription rate. The wide array of ethnic groups with distinct genetic predispositions and socioeconomic backgrounds amongst the Hispanic population makes it challenging to draw overarching conclusions. A study of over 16,000 Hispanic patients confirmed an association between lower socioeconomic class, Spanish language preference and other cardiovascular risk factors with higher rates of dyslipidemia [13]. Furthermore, Cubans and South Americans tend to have higher rates of dyslipidemia, while Dominicans and Puerto Ricans have higher statin adherence and awareness of their hyperlipidemia [13,14,15].

Statin underprescription is complex and a multifaceted approach and the comprehension of these barriers is needed to address this healthcare burden. Physician bias, low health literacy, poor follow-up, high rate of problem-based versus preventative visits and language barriers account for some of these challenges. Although our data were limited to a single center, this study reflects disparities in statin prescription patterns in a population where most patients represent minority communities. It is known that these patients face educational and socioeconomic challenges that can be barriers to statin prescription. Although our medical record system does not document educational level, our main patient population is underserved minorities. These socioeconomic factors can influence prescription patterns due to patient preference or hesitancy to statin use. Since the main focus of our current study was to assess prescription patterns of physicians based on adherence to guidelines, any documented patient preference against statin prescription was used as a patient exclusion criterion. Additional studies can further assess the impact of educational and socioeconomic level on appropriate statin use in this patient population. Our data offer a unique insight into statin prescription patterns whereby, even in a minority underserved setting, black patients continue to experience undertreatment. A follow-up quality improvement study with formal educational interventions is currently underway in order to address this disparity in statin prescription and to assess improvement in practice patterns over time.

## 5. Conclusions

The primary and secondary prevention of ASCVD is dependent on optimal guideline-directed pharmacotherapy. The AHA/ACC cholesterol guidelines emphasize proper screening and treatment monitoring to ensure that lipid-lowering agents are appropriately dosed and adjusted. Our population of patients provides a unique insight into healthcare disparities in statin prescription patterns. Although our center has a plurality of black patients, our data have similarities to other studies where black patients did not comprise such a large percentage of the population and also provides interesting results on statin prescription patterns in Hispanics patients relative to black patients. Our results suggest that certain known socioeconomic health disparities fail to correct when black patients are no longer a minority group relative to other races. Because multiple systemic barriers exist to providing optimal care, comprehensive assessment of barriers to appropriate statin prescription, particularly in minorities and underserved populations, is paramount. In addition, emphasis needs to be placed on cardiovascular risk stratification in younger patients based on the presence of risk enhancers and close LDL-C monitoring to evaluate treatment adherence and efficacy. Moving forward, in order to improve statin prescription patterns and ultimately better cardiovascular outcomes in minority black and Hispanic populations, we need larger studies to systematically understand the specific challenges surrounding these communities.

## Figures and Tables

**Table 1:** Patient characteristics.

	Total (*n* = 464)	Statin Prescribed (*n* = 381)	Statin Not Prescribed (*n* = 83)	*p*
Demographics				
Age, years	61.0 (10.4)	61.8 (10.5)	57.2 (9.1)	0.0001
Males, *n* (%)	214 (46.1)	177 (46.5)	37 (44.6)	
Race/Ethnicity, *n* (%)				0.12
Black or African American	219 (47.2)	169 (44.4)	50 (60.2)	
Hispanic or Latino	212 (45.7)	184 (48.3)	28 (33.7)	
White	23 (5.0)	19 (5.0)	4 (4.8)	
Asian	9 (1.9)	8 (2.1)	1 (1.2)	
Native Hawaiian/Pacific Islander	1 (0.2)	1 (0.3)	0 (0.0)	
Primary Language, *n* (%)				0.500
English	284 (61.2)	227 (59.6)	57 (68.7)	
Spanish	130 (28.0)	111 (29.1)	19 (22.9)	
Portuguese	31 (6.7)	26 (6.8)	5 (6.0)	
Other	15 (3.2)	14 (3.7)	1 (1.2)	
French Creole	4 (0.9)	3 (0.8)	1 (1.2)	
Translator utilized/Physician fluent	127 (27.4)	107 (28.1)	20 (24.1)	0.656
Uninsured, *n* (%)	254 (54.7)	207 (54.3)	47 (56.6)	0.796
BMI, kg/m^2^	29.4 [25.7–33.4]	29.4 [25.8–33.3]	29.0 [25.2–35.1]	0.875
Comorbidities, *n* (%)				
Hypertension	376 (81.0)	320 (84.0)	56 (67.5)	0.001
Systolic BP	142.0 (65.3)	142.1 (71.5)	141.7 (21.0)	0.956
Diastolic BP	73.7 (12.0)	73.0 (11.5)	77.0 (13.5)	0.006
Diabetes Mellitus	209 (45.0)	209 (45.0)	209 (45.0)	0.0001
Hemoglobin A1c	7.2 [6.5–8.9]	7.2 [6.5–8.9]	7.2 [6.5–8.9]	0.063
Chronic kidney disease	71 (15.3)	67 (17.6)	4 (4.8)	0.006
Congestive heart failure	60 (12.9)	56 (14.7)	4 (4.8)	0.024
Coronary artery disease	67 (14.4)	67 (17.6)	0 (0.0)	0.0001
History of MI	32 (6.9)	31 (8.2)	1 (1.2)	0.043
History of PCI	30 (6.5)	30 (7.9)	0 (0.0)	0.017
History of CABG	15 (3.2)	15 (3.9)	0 (0.0)	0.135
Cerebrovascular disease	68 (14.7)	65 (17.1)	3 (3.6)	0.003
Peripheral artery disease	13 (2.8)	12 (3.1)	1 (1.2)	0.545
Current smoker	83 (17.9)	63 (16.5)	20 (24.1)	0.206
Lipid profile, mg/dL				
LDL-C	87.0 [64.0–120.0]	84.0 [62.5–121.0]	93.0 [72.0–112.5]	0.339
HDL-C	50.0 [41.0–59.0]	49.0 [40.0–58.0]	52.0 [43.8–63.8]	0.048
Triglycerides	103.0 [78.0–149.0]	104.5 [80.2–150.0]	95.5 [69.5–135.8]	0.056
Statin Indications, *n* (%)				0.0001
Very high-risk ASCVD	76 (16.4)	73 (19.2)	3 (3.6)	
Clinical ASCVD	48 (10.3)	46 (12.1)	2 (2.4)	
10-year ASCVD risk >7.5	185 (39.9)	125 (32.8)	60 (72.3)	
Diabetes, age 40–75	145 (31.2)	127 (33.3)	18 (21.7)	
LDL-C >190mg/dL	10 (2.2)	10 (2.6)	0 (0.0)	

Values represent mean ± standard deviation, median [IQR 25th–75th percentiles] or number (%). Bold values indicate statistical significance (*p* < 0.05). CABG = coronary artery bypass grafting; HDL-C = high-density lipoprotein cholesterol; LDL-C = low-density lipoprotein cholesterol; MI = myocardial infarction; PCI = percutaneous coronary intervention.

## Abbreviations

ASCVD	atherosclerotic cardiovascular disease
CVD	cardiovascular disease
ACC	American College of Cardiology
AHA	American Heart Association (only 5–6 abbreviations)
LLT	lipid lowering therapy
LDL	low density lipoprotein
